# A Detailed Examination of Retroperitoneal Undifferentiated Pleomorphic Sarcoma: A Case Report and Review of the Existing Literature

**DOI:** 10.3390/jcm13133684

**Published:** 2024-06-25

**Authors:** Goran Balovic, Bojana S. Stojanovic, Dragce Radovanovic, Dejan Lazic, Milena Ilic, Ivan Jovanovic, Dejan Svilar, Vesna Stankovic, Jelena Sibalija Balovic, Bojana Simovic Markovic, Milica Dimitrijevic Stojanovic, Dalibor Jovanovic, Bojan Stojanovic

**Affiliations:** 1 Department of Surgery, Faculty of Medical Sciences, University of Kragujevac, 34000 Kragujevac, Serbia; gbalovic@gmail.com (G.B.); drakce_5@hotmail.com (D.R.); dlazic.kg@gmail.com (D.L.); bojan.stojanovic01@gmail.com (B.S.); 2 Department of Pathophysiology, Faculty of Medical Sciences, University of Kragujevac, 34000 Kragujevac, Serbia; 3 Center for Molecular Medicine and Stem Cell Research, Faculty of Medical Sciences, University of Kragujevac, 34000 Kragujevac, Serbia; ivanjovanovic77@gmail.com (I.J.); bojana.simovic@gmail.com (B.S.M.); 4 Department of Pathology, Faculty of Medical Sciences, University of Kragujevac, 34000 Kragujevac, Serbia; lena.ilic@gmail.com (M.I.); wesna.stankovic@gmail.com (V.S.); dalekg84@gmail.com (D.J.); 5 Department of Radiology, Faculty of Medical Sciences, University of Kragujevac, 34000 Kragujevac, Serbia; swilar@gmail.com; 6 Department of Pediatrics, University Clinical Center Kragujevac, 34000 Kragujevac, Serbia; jmbalovic@gmail.com

**Keywords:** retroperitoneal undifferentiated pleomorphic sarcoma, diagnostic challenges, therapeutic strategies, molecular pathogenesis, multidisciplinary treatment, targeted therapy

## Abstract

This detailed review focuses on retroperitoneal undifferentiated pleomorphic sarcoma (UPS), a particularly aggressive soft-tissue sarcoma that poses unique diagnostic and therapeutic challenges due to its rarity and complex presentation. By documenting a new case of retroperitoneal UPS and conducting a comprehensive review of all known cases, this article aims to expand the existing body of knowledge on the epidemiology, molecular pathogenesis, and treatment strategies associated with this rare disease. The complexity of diagnosing UPS is emphasized given that it rarely occurs in the retroperitoneal space and its histological and molecular complexity often complicates its recognition. This review highlights the need for specialized diagnostic approaches, including advanced imaging techniques and histopathological studies, to accurately diagnose and stage the disease. In terms of treatment, this paper advocates a multidisciplinary approach that combines surgery, radiotherapy and chemotherapy and tailors it to individual patients to optimize treatment outcomes. This review highlights case studies that illustrate the effectiveness of surgical intervention in the treatment of these tumors and emphasize the importance of achieving clear surgical margins to prevent recurrence. Furthermore, this review discusses the potential of new molecular targets and the need for innovative therapies that could bring new hope to patients affected by this challenging sarcoma.

## 1. Introduction

Undifferentiated pleomorphic sarcoma (UPS), formerly known as malignant fibrous histiocytoma (MFH), is a rare and aggressive type of soft-tissue sarcoma (STS) that represents approximately 1–2% of all solid tumors [1]. Although UPS commonly presents in the extremities, its occurrence in the retroperitoneal space is rare, posing significant diagnostic and therapeutic challenges [2]. Retroperitoneal UPS is an oncologically complex pathology due to its infrequent manifestation and aggressive nature, which complicates both diagnosis and management [3]. Distinguished by its specific histological and molecular profiles, UPS requires specialized diagnostic approaches and individualized therapeutic strategies.

The purpose of this article is to extend the existing literature by documenting new cases of retroperitoneal UPS, reviewing all known cases, and discussing broad aspects of the disease, including its epidemiology, pathogenesis, and current treatment strategies. Through a comprehensive analysis of clinical and molecular features, diagnostic hurdles, and treatment options, this report aims to highlight the need for a multidisciplinary approach to the treatment of this complex sarcoma, ultimately aiming to improve patient outcomes and promote the advancement of targeted therapies.

## 2. Case Presentation

A 60-year-old male patient was admitted to the Department of Surgery in our hospital. He presented with nonspecific symptoms, including abdominal pain, bloating, progressive loss of appetite, and significant weight loss of approximately 10 kg over the past two months. His medical history was characterized by a persistent dry and irritating cough, treatment by a pulmonologist, and previous COVID-19 infection. He denied any history of chronic illness or injuries to the chest and abdomen. Abdominal physical examination revealed no obvious abnormalities. Laboratory tests revealed anemia (hemoglobin 109 g/L, normal range 130–175 g/L; hematocrit 34.1%, normal range 41.5–53%), an increased erythrocyte sedimentation rate, and a significantly elevated C-reactive protein level (91.5 mg/L, normal range 0–5 mg/L).

**Figure 1:**
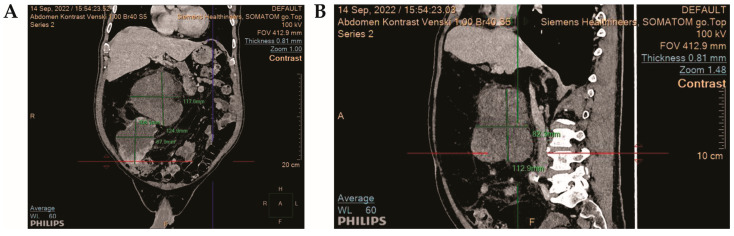
**Imaging of Retroperitoneal Undifferentiated Pleomorphic Sarcoma:** (**A**) shows the coronal view, highlighting the tumor’s extent and relationship with surrounding anatomical structures. (**B**) presents the sagittal view, providing insights into the depth and infiltration of the tumor into adjacent tissues.

Initial abdominal complaints and significant weight loss this patient prompted advanced diagnostic evaluations beyond the normal chest X-ray. Abdominal ultrasonography revealed a large transsonic mass in the retroperitoneum, requiring further evaluation with computed tomography (CT). The CT scan of the abdomen and pelvis displayed a large intra-abdominal tumor measuring approximately 21 × 16 × 22 cm^3^ with lipomatous components and three heterogeneous solid lesions within. This mass had displaced right abdominal organs to the left and affected the kidneys, vascular structures, and small intestinal loops (Figure 1).

**Figure 2:**
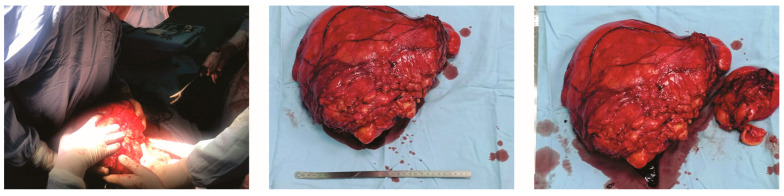
**Intraoperative Gross Observation of Undifferentiated Pleomorphic Sarcoma:** This image showcases the undifferentiated pleomorphic sarcoma, measuring 325 × 260 × 110 mm^3^ and weighing 4400 g, as observed during surgery.

Given the clinical presentation and imaging findings, the decision was made to proceed with surgical exploration and possible resection of the mass. The patient underwent a medial laparotomy under general anesthesia, revealing an extensive lipomatous tumor occupying the entire medial portion of the abdomen, causing significant displacement and compression of the abdominal organs. The mass was adherent to the duodenum and the lower pole of the left kidney, necessitating meticulous adhesiolysis to detach it. Following successful separation, the tumor was carefully excised with clear margins, ensuring the preservation of adjacent structures. The excised tumor appeared as a solid, bloody, yellowish mass, encapsulated by a clearly visible perirenal fat layer, as illustrated in Figure 2.

The histopathological examination of the excised tumor revealed a gross morphology of a pinkish and lobulated mass. The cut surface of the tissue revealed a predominantly yellowish color interspersed with multilocular cystic structures, the largest of which was 130 mm in diameter and filled with partially coagulated, yellowish-brown fluid. Adjacent to this was a solid nodular lesion, approximately 90 mm in length, characterized by a firm, whitish appearance with a bacon-like texture. Furthermore, a separate area of the tumor, 200 mm in diameter, exhibited a greyish-brown coloration, containing multiple smaller cysts ranging from 5 to 25 mm in diameter.

**Figure 3:**
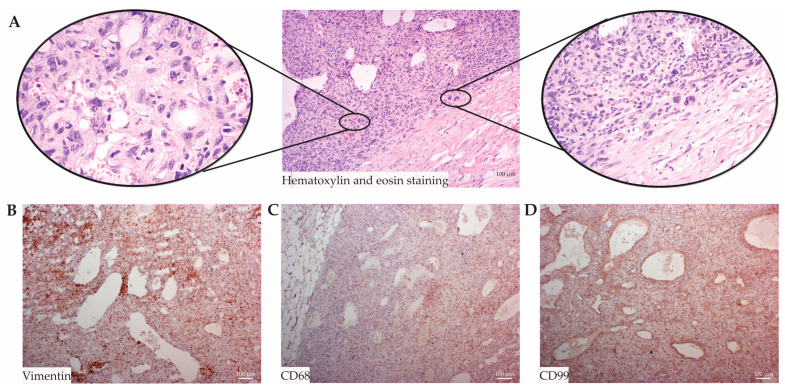
**Histological and Immunohistochemical Staining of Undifferentiated Pleomorphic Sarcoma:** This figure illustrates various staining techniques used to characterize the tumor. (**A**) Hematoxylin and Eosin (H&E) staining shows the general cellular morphology of the tumor. (**B**) Immunohistochemical staining for Vimentin highlights mesenchymal cells, indicative of the tumor’s origin. (**C**) CD68 staining identifies macrophage populations within the tumor, suggesting an immune presence. (**D**) CD99 staining detects surface protein expression that may be relevant to the tumor’s oncogenic processes. The areas within the rectangles are magnified at ×20, while the areas within the circles are magnified at ×40.

A histopathological examination of the tumor tissue provides detailed features indicating a highly complex and heterogeneous tumor (Figure 3A). The tissue is composed primarily of adipose tissue with rapid expansion of the tumor cells confined by a compressed, lamellar-like hypocellular connective tissue framework. Within this structure, isolated tumor cells exhibited characteristics suggestive of rhabdomyoplastic and neurogenous differentiation, evident across various parts of the tumor. Extensive areas of necrosis and hemorrhage were noted, contributing to the tumor’s morphological diversity. The histomorphological spectrum was markedly heterogeneous, displaying multiple differentiation pathways and organizational patterns. This included areas with irregular storiform arrangements dominated by polymorphic histiocyte-like cells with bubble-like cytoplasm and a range of nuclear atypia including mononuclear, multinucleated, and bizarre nuclei. The tumor displayed uneven cellularity, alternating between moderate and hypercellular zones, interspersed with myxoid transformations and a consistent inflammatory infiltrate. The vascular architecture within the tumor was notable for “hemangiopericytoma-like” spaces, alongside “osteoid and collagen-like” foci and segments showing a pronounced epithelioid cellular morphology. Multinucleated giant cells with bizarre nuclei were predominantly observed in certain sections. Notably, there was no evidence of lymphovascular invasion or nodal involvement. Immunohistochemical analysis showed tumor cells expressing vimentin (Figure 3B), CD68 (Figure 3C), CD99 (Figure 3D), and CD10 focally, while negative for SMA, CD34, S100, melan-A, CD57, CD56, p53, LCA, CD117, DOG1, AE1/AE3, and Ki-67, with a proliferative index of approximately 30%. The final pathologic diagnosis confirmed the tumor as an unclassified, undifferentiated pleomorphic sarcoma.

The patient’s postoperative recovery was uneventful, and he was discharged 7 days after the surgical intervention. He was enrolled in a surveillance program with regular follow-up visits, including an initial CT scan of the chest and abdomen, which showed normal results. However, during a routine follow-up one year post-surgery, a CT scan of the chest detected a nodular formation in the upper right lobe, suggestive of pulmonary metastasis, while the CT scan of the abdomen was normal, with no recurrence of the disease. The patient subsequently underwent stereotactic radiosurgery to target these metastases. This treatment was well-tolerated, and one year after the intervention, follow-up imaging demonstrated a significant reduction in the size of the lung nodules, indicating a positive response to the intervention.

## 3. Discussion

Soft-tissue sarcomas represent a rare and diverse group of mesenchymal origin tumors, accounting for about 1% of adult malignancies, with undifferentiated pleomorphic sarcoma standing out due to its high malignancy and poor prognosis [4]. Initially identified as malignant fibrous histiocytoma, UPS has undergone various nomenclatural changes and is currently recognized for its pleomorphic, often undifferentiated cellular characteristics, making it the most prevalent soft-tissue tumor in adults, occurring in 20% of cases [5]. This tumor type predominantly affects middle-aged and elderly males and is more commonly found in the extremities than in the retroperitoneal space [6,7,8]. Despite its low incidence—increasing from 3 cases per 100,000 people in 2013 to three 3 per 45,000 currently—UPS demonstrates a worrying trend towards a gradual increase in occurrence [6,9]. The primary sites for metastatic spread are typically the lungs, as demonstrated in our case report, and less frequently, the liver [4,10].

### 3.1. Histogenetic Evolution of Pleomorphic Undifferentiated Sarcoma

The histogenetic origins of UPS, previously recognized as MFH, have long been a subject of controversy [11]. Initially defined in 1964 by O’bren and Stout, the tumor was characterized by cellular indicators suggestive of both mesenchymal and mononuclear phagocytic system origins [12,13]. The nomenclature and classification of these tumors have evolved significantly over the decades. In 1987, Ghandur-Mnaymanch proposed renaming it as fibrous histiocytic sarcoma, emphasizing the tumor’s dual behavior resembling both sarcoma and lymphoma [14]. This recommendation was a step towards refining the understanding of the tumor’s biological behavior and its classification under a more descriptive term. More recently, the World Health Organization (WHO) has made further adjustments by officially adopting the term pleomorphic undifferentiated sarcoma in the medical literature and discontinuing the use of malignant fibrous histiocytoma [15]. It was recognized that MFH represents a diverse group of poorly differentiated neoplasms. The theories regarding the origins of UPS included suggestions that it could originate from a primitive pluripotential mesenchymal cell capable of varying degrees of differentiation, or that it might represent a nonspecific category encompassing a range of poorly differentiated neoplasms [4,16].

### 3.2. Risk Factors Associated with Undifferentiated Pleomorphic Sarcoma

Undifferentiated pleomorphic sarcoma is associated with several established risk factors that contribute to its development. Smoking has been identified as a significant risk factor, with an odds ratio of 2.05, indicating that smokers are over twice as likely to develop UPS compared to non-smokers [17]. Additionally, a considerable portion of UPS patients, more than 30%, report a family history of the disease, suggesting a potential genetic predisposition or hereditary component in its pathogenesis [17,18,19]. Another notable risk factor is exposure to radiation therapy, which has been linked to the development of a specific subtype of UPS, known as radiation-associated undifferentiated pleomorphic sarcoma (RA-UPS). This subtype affects approximately 3–5% of patients who have undergone radiation treatment [20].

### 3.3. Anatomical Localization of Undifferentiated Pleomorphic Sarcoma

Undifferentiated pleomorphic sarcoma exhibits a distinct preference for anatomical localization, predominantly affecting the extremities, where it is most frequently diagnosed [8]. Approximately 50% of all UPS cases are found in the lower extremities, and another 20% in the upper extremities, predominantly affecting middle-aged to elderly males [21,22]. Conversely, the occurrence of UPS in the retroperitoneum is notably rare, with our case representing only the eighth documented instance [6,13,23,24,25,26,27].

### 3.4. Molecular Pathogenesis of Undifferentiated Pleomorphic Sarcoma

The molecular pathogenesis of UPS is driven by a complex array of genetic and epigenetic changes that influence tumor behavior including proliferation, invasion, and migration [28]. This complexity includes alterations such as mutations, deletions, and epigenetic modifications which play pivotal roles in the development and progression of UPS [28]. Notably, a range of “cancer driver genes” such as tumor protein p53 (TP53), alpha-thalassemia/mental retardation syndrome X-linked (ATRX), H3 histone family member 3A (H3F3A), and zinc finger homeobox 3 (ZFHX3), among others, have been identified as critical to the onset and advancement of the disease [29,30]. The inactivation of tumor suppressor genes is a frequent occurrence, with mutations in TP53 leading to the overexpression of p53 [30]. This condition is commonly linked with increased tumor recurrence, metastasis, and an overall poor prognosis. Conversely, the absence of TP53 expression correlates with the activation of S-phase kinase-associated protein 2 (Skp2), enhancing cell proliferation through the degradation of cyclin-dependent kinase inhibitor 1A (p21) and cyclin-dependent kinase inhibitor 1B (p27) [31].

Additionally, the role of cellular senescence in UPS is highlighted by the homozygous deletion of cyclin-dependent kinase inhibitor 2A (p16INK4a), although it appears that p16 may not act as a predominant barrier to senescence in some UPS subtypes [28]. From an epigenetic perspective, significant modifications include the methylation of sites around integrin subunit alpha 10 (ITGA10) and protein phosphatase 2 regulatory subunit B (PPP2R2B), which are upstream regulators of the Akt/mTOR (protein kinase B/mammalian target of rapamycin) signaling pathway. These epigenetic changes affect the expression of downstream signaling components such as triple functional domain protein (TRIO) and rapamycin-insensitive companion of mTOR (RICTOR), thus activating the RAC/PAK (Ras-related C3 botulinum toxin substrate/p21-activated kinase) and Akt/mTOR pathways that are crucial for UPS cell survival [28,32].

### 3.5. Signaling Pathways and Molecular Drivers in Undifferentiated Pleomorphic Sarcoma

In undifferentiated pleomorphic sarcoma, a diverse array of signaling pathways plays a crucial role in the oncogenic process, with notable involvement of the phosphoinositide 3-kinase/protein kinase B/mammalian target of rapamycin (PI3K/Akt/mTOR) and Hippo signaling pathways [33,34,35]. These pathways have been frequently implicated in the disease’s pathogenesis due to their influence on cell proliferation, invasion, and migration. Additionally, the hepatocyte growth factor/MET (HGF/MET) pathway, mediated by the transmembrane tyrosine kinase receptor MET, plays a critical role in UPS. Pathological studies have demonstrated that gene amplification of MET and receptor overexpression significantly promote cellular behaviors associated with tumor aggressiveness [36].

The aberrant activation of these pathways in UPS is often compounded by independent mechanisms such as receptor overexpression, altered transcription, or post-translational modifications, which underscore the complexity of UPS pathogenesis. For instance, microRNA-152 (miR-152) downregulation has been linked to increased tyrosine kinase receptor (TKR) mRNA and protein levels, indicating a pivotal role in sarcomagenesis [37]. Similarly, dysregulation of the Rho/ROCK signaling pathway, modified by miR-138, has been associated with metastatic tendencies in UPS due to the specific overexpression of miR-138 in metastatic cells [38]. Furthermore, signaling interconnections are evident, as seen with the insulin-like growth factor 1 receptor (IGF1R), which acts as a common upstream regulator for both the PI3K/mTOR and RAS/mitogen-activated protein kinase (MAPK) pathways, suggesting a compensatory activation that persists even with the inhibition of a single pathway [39].

Moreover, components such as heat shock protein 90 (HSP90) and signal transducer and activator of transcription 3 (STAT3) influence cellular invasiveness and viability through the phosphorylation of pathway components, with phosphorylated STAT3 being notably associated with poor prognosis. The complex interaction between these molecular pathways illustrates the intricate network of cellular signaling involved in UPS.

### 3.6. The Role of the Peritumoral Microenvironment in Undifferentiated Pleomorphic Sarcoma

The tumor microenvironment plays a critical and complex role in the progression of UPS, particularly through its inflammatory components [28]. Within this microenvironment, tumor-associated macrophages (TAMs) are prominent players, known for their protumoral activities [40]. These macrophages produce significant levels of cytokines such as transforming growth factor-beta (TGFβ) and interleukin-6 (IL6), which activate downstream signaling pathways leading to enhanced tumor cell proliferation, migration, and invasion [40]. The proportion of TAMs within the tumor has been identified as a prognostic factor in UPS, indicating their significant impact on tumor behavior and patient outcomes [41].

Additionally, the infiltration of dendritic cells and neutrophils into the tumor microenvironment correlates with key prognostic indicators including recurrence-free survival (RFS) and disease-specific survival (DSS) [42]. These findings highlight an inflammatory milieu surrounding UPS, characterized by a high expression of antigen presentation genes and regulatory T-cell genes [43]. This genetic alteration enables robust oligoclonal T-cell infiltration, which in turn upregulates programmed death-ligand 1 (PD-L1) and other inhibitory ligands, thereby helping tumors evade immune surveillance [41].

### 3.7. Clinical Presentation of Retroperitoneal Undifferentiated Pleomorphic Sarcoma

Retroperitoneal UPS generally manifests in the sixth to seventh decades of life with symptoms that are indirect and related to the tumor’s impact on surrounding tissues [6]. The symptoms vary based on the tumor’s size and location, commonly including nonspecific abdominal pain, weight loss, and anemia, as demonstrated in our case [6]. These symptoms are often due to tumor compression or its metabolic demands. Additionally, some patients may present with systemic symptoms like fever or difficulty in urination, as seen in a minority of cases [6]. Moreover, respiratory symptoms can occur in cases where the tumor has metastasized to the lungs [44].

### 3.8. Diagnostic Challenges and Imaging Features of Undifferentiated Pleomorphic Sarcoma

Diagnosing undifferentiated pleomorphic sarcoma poses significant challenges due to its rarity and the nonspecific nature of its imaging characteristics. The radiological literature on UPS is limited, and the imaging findings are typically non-distinct, often necessitating the use of exclusionary diagnostic methods. While imaging modalities such as computed tomography (CT) and magnetic resonance imaging (MRI) are pivotal, they generally reveal features that could be mistaken for other types of soft-tissue sarcomas [45]. For instance, well-differentiated liposarcomas usually show adipose tissue within the tumor, whereas some poorly differentiated liposarcomas exhibit calcifications. In contrast, smooth muscle sarcomas might display vascular invasion and hemorrhagic necrosis [45,46]. Advanced imaging techniques, such as CT and MRI, play pivotal roles in the accurate diagnosis and staging of retroperitoneal UPS.

Specifically for UPS, CT imaging may show a density similar to adjacent muscle with heterogeneous lower density areas indicative of hemorrhage, necrosis, or myxoid material, and the soft-tissue component often enhances after contrast administration [47]. MRI, considered the superior modality for assessing soft-tissue sarcomas, effectively stages these tumors and provides detailed characterization. UPS typically appears well circumscribed, located within or adjacent to muscle tissues and may exert a mass effect on surrounding structures due to their size. On T1-weighted images, these tumors usually present with intermediate to low signal intensity, similar to muscle, with heterogeneity noted if hemorrhage, calcification, or necrosis is present. T2-weighted images typically show intermediate to high signal intensity with similar heterogeneity [48].

A distinctive feature in CT, known as the “tail sign”—a curvilinear signal extension from the mass—is sometimes observed in cases of infiltrative UPS. This sign can serve as a valuable diagnostic marker and also plays a crucial role in surgical planning. For optimal surgical outcomes, imaging is essential to establish appropriate surgical margins, typically set 2–3 cm beyond the tumor infiltration displayed on imaging. This approach ensures that the resection margins are sufficient to manage the local spread of the tumor effectively, thereby reducing the risk of recurrence and improving prognosis [49,50].

The accurate staging of UPS is essential for optimal treatment planning and prognostication. Advanced imaging techniques provide critical information on the tumor’s size, extent, and relationship with adjacent structures. This information is vital for determining the stage of the disease and guiding the surgical approach. For optimal surgical outcomes, imaging is essential to establish appropriate surgical margins. Furthermore, advanced imaging techniques are indispensable for detecting metastasis, which is crucial for staging and treatment planning. UPS has a propensity to metastasize to the lungs, liver, and other distant sites. CT scans of the chest and abdomen, as well as MRI or PET scans, are used to identify metastatic lesions [45,46,48].

### 3.9. Histopathological Profile of Undifferentiated Pleomorphic Sarcoma

The histopathological examination of UPS reveals a complex and highly variable tumor architecture, which is critical for its diagnosis and subsequent management. The assessment of retroperitoneal mesenchymal tumors, including UPS, remains a challenge due to their morphology frequently overlapping with several other tumors arising in the retroperitoneum. The World Health Organization’s 5th edition characterizes UPS by a predominance of spindle cell, pleomorphic, epithelioid, and round cell morphologies, typically presenting with a high-grade morphology. The gross pathological features of UPS usually include a well-circumscribed, multilobulated mass with a heterogeneous cut surface that appears gray-tan and is firm and fleshy. Necrosis and hemorrhage within the tumor mass are commonly observed, indicating the aggressive nature of the tumor [51]. The frozen section often reveals a storiform, fascicular, or patternless arrangement of highly atypical cells, including spindled, epithelioid, or pleomorphic types. This section is also notable for abundant mitotic figures and frequent areas of coagulative necrosis, further underscoring the tumor’s high-grade characteristics. Microscopically, UPS demonstrates similar architectural features with frequent bizarre multinucleated tumor giant cells and a cytoplasm that ranges from amphophilic to palely eosinophilic. The nuclei are often hyperchromatic or vesicular, and the presence of abundant mitoses reflects the high proliferative activity of the tumor [51].

### 3.10. Immunohistochemistry, Molecular Diagnostics, and WHO Criteria in the Diagnosis of Undifferentiated Pleomorphic Sarcoma

The role of immunohistochemistry in diagnosing UPS is primarily supportive, used to exclude other pleomorphic tumors rather than to confirm UPS directly [52]. Commonly, UPS may show variable expression of markers such as CD34, smooth muscle actin (SMA), and CD68. However, the focal expression of these markers is nonspecific and does not indicate a particular line of differentiation, which aligns with the tumor’s undifferentiated nature [53].

On a molecular level, UPS is characterized by numerous and varied chromosomal aberrations. Frequent numerical and structural variations suggest a high degree of genetic instability [28]. Notably, losses at chromosomal regions 13q12-q14 and 13q21 are common, hinting at the possible presence of tumor suppressor genes in these areas that, when lost, contribute to tumorigenesis [54]. Integrated genomic studies have also shown that UPS and myxofibrosarcoma share similar molecular profiles, further complicating the diagnostic process [28]. Additionally, PRDM10 fusions, found in approximately 5% of cases, highlight the diversity within the molecular landscape of UPS [55].

According to the World Health Organization’s 5th edition criteria, the diagnosis of UPS is largely one of exclusion [5]. This approach is necessitated by the tumor’s high-grade morphology and the typical absence of specific morphological and immunohistochemical differentiation features. UPS is defined by the presence of atypical spindle cell, pleomorphic, epithelioid, and round cells, with a high-grade morphology prevalent in the majority of cases. The diagnosis also requires an absence of distinctive gene fusions, particularly for undifferentiated round cell sarcomas, a subtype often seen in younger patients [5,56].

### 3.11. Biomarkers in Retroperitoneal Undifferentiated Pleomorphic Sarcoma

Identifying reliable biomarkers for retroperitoneal UPS is essential for improving diagnosis, prognosis, and treatment strategies. To date, the identified biomarkers include tumor-infiltrating macrophages (TIMs), alterations in the TP53 gene, and specific microRNAs.

Retroperitoneal UPS is more frequently infiltrated by macrophages than by lymphocytes, indicating that TIMs play a significant role in the tumor microenvironment and could serve as valuable biomarkers [57]. The TP53 gene, which encodes the p53 protein, shows frequent alterations in UPS. This protein regulates cell cycle arrest, apoptosis, and metabolism, making its alterations crucial for understanding UPS pathogenesis and potential therapeutic targets [58].

Furthermore, several microRNAs are dysregulated in UPS. miR-126, miR-223, miR-451, and miR-1274b are significantly upregulated, while miR-100, miR-886-3p, miR-1260, miR-1274a, and miR-1274b are significantly downregulated compared to control mesenchymal stem cell lines. Additionally, miR-199-5p and miR-320a can differentiate between undifferentiated pleomorphic sarcoma and leiomyosarcoma, underscoring their diagnostic potential [59].

### 3.12. Multidisciplinary Approach in the Treatment of Retroperitoneal UPS

A multidisciplinary approach is crucial for the effective treatment of retroperitoneal UPS due to the complex nature and rarity of the disease. This approach involves the collaboration of various specialists, including surgical oncologists, medical oncologists, radiologists, pathologists, and radiation oncologists.

Surgical resection with clear margins is the cornerstone of treatment for localized disease and often requires preoperative planning and postoperative care involving radiologists and surgical oncologists. Medical oncologists contribute by determining the appropriate chemotherapy regimens, particularly in cases with high-risk features or metastatic disease. Radiation oncologists may provide preoperative or postoperative radiotherapy to reduce the risk of local recurrence. Pathologists play a critical role in accurately diagnosing the tumor and identifying relevant biomarkers for targeted therapy.

### 3.13. Surgical Treatment of Undifferentiated Pleomorphic Sarcoma

Surgery remains the primary treatment for UPS, with radical resection aimed at achieving negative surgical margins being pivotal. This approach is essential due to the high risk of local recurrence and metastasis associated with residual tumor presence [28]. The infiltrative growth pattern of UPS presents significant challenges in achieving clear margins, complicating complete excision and adversely affecting long-term outcomes [60].

The importance of surgical margins in managing UPS is emphasized across various studies, which propose different thresholds for what constitutes an adequate margin. For example, one retrospective study advocates for a minimum resection margin of 10 mm to optimize local control, while another suggests that a microscopic margin of at least 2.5 cm is necessary to achieve a 90% local control rate over five years [61,62]. Furthermore, the Union for International Cancer Control classification recommends an R0 resection (minimal resection margin > 1 mm) as ideal, indicating that wider margins correlate with better local control rates compared to narrower margins [63].

### 3.14. The Role of Radiation Therapy in the Management of Undifferentiated Pleomorphic Sarcoma

Radiation therapy plays a significant role in the treatment of UPS, particularly for tumors that are localized and have not yet infiltrated deeply. It is most effective in UPS cases located in the extremities and when the tumors are superficial [64]. Radiation can induce increased antigen expression at the tumor site, enhance immune cell infiltration and antigen cross-presentation, and modify the tumor microenvironment. These changes can inhibit tumor cell proliferation, thereby augmenting the therapeutic effect [65].

However, the benefits of radiation therapy diminish in UPS with infiltrative metastases, where it has not shown significant effectiveness [66]. This limitation was evident in our patient, whose tumor was located in the retroperitoneum: a site where radiation therapy often yields less favorable outcomes, reflecting findings from previous studies [6].

The timing of radiation therapy also influences its effectiveness. Perioperative radiation, particularly when administered preoperatively, has been associated with prolonged survival times in UPS patients [67]. This approach may help reduce the tumor size or limit its spread, making surgical resection more feasible and potentially more effective. Despite its benefits, radiation therapy is not without risks. One of the severe side effects includes the development of radiation-associated undifferentiated pleomorphic sarcomas [68].

### 3.15. Adjuvant Therapy for Undifferentiated Pleomorphic Sarcoma

Chemotherapy has shown variable success in the treatment of UPS, particularly for deep-seated tumors within several parenchymal organs and in cases of radiation-associated UPS, where it often outperforms radiation therapy [69]. The most commonly used chemotherapy regimen includes anthracyclines plus ifosfamide (A + I), recognized for extending survival in certain patient subsets. However, the effectiveness of this regimen and others, such as the combination of adriamycin with cyclophosphamide, varies due to the tumor’s high heterogeneity [70]. Studies like those conducted by Young et al. demonstrate a higher response rate and improved overall survival with combined agent chemotherapy compared to single-agent regimens [71]. Despite these findings, the overall clinical response to chemotherapy remains limited, and secondary tumor side effects are generally less severe than those associated with radiation [72].

The search for more effective treatments has led to the exploration of novel agents such as Trabectedin, which has shown promise in UPS after the failure of standard regimens like A + I [73]. The application of combination chemotherapy, despite its increased toxicity, might be justified in younger, fit patients who can tolerate more aggressive treatments. For instance, randomization trials comparing Gemcitabine plus docetaxel (G + D) against A + I found no superiority in the former, suggesting that while new combinations are being tested, they do not always yield better outcomes [74].

The timing and dosing of chemotherapy also present challenges. A Japanese trial (JCOG0304) noted limited patient benefits from perioperative chemotherapy due to significant hematological toxicities [75]. Similarly, a comparison of preoperative three-cycle and perioperative five-cycle regimens of epirubicin-ifosfamide revealed comparable outcomes, suggesting that if adjuvant chemotherapy is necessary, it might be more effectively administered preoperatively and limited to three cycles [76].

### 3.16. Targeted Therapy in the Management of Undifferentiated Pleomorphic Sarcoma

The aggressive nature of UPS, characterized by a low tumor mutational burden and high copy number alterations, poses significant challenges in treatment [77]. Recent advances have highlighted the programmed death-1 (PD-1) and programmed death-ligand 1 (PD-L1) pathways as crucial targets in the tumor immune evasion mechanism [78]. Targeted therapy focusing on these markers, such as PD-1 inhibitors, has shown potential in improving patient outcomes through enhancing immune cell infiltration and modifying the tumor microenvironment [79].

Studies by YangYou et al. and Zhichao Tian et al. have demonstrated the effectiveness of combining anti-angiogenesis inhibitors or paclitaxel with PD-1 inhibitors, presenting a promising strategy for enhancing therapeutic efficacy in UPS [80,81]. However, the response to PD-1 inhibitors varies, with some patients experiencing poor outcomes, which underscores the complexity of immune response in UPS treatment.

The modulation of tyrosine kinase receptors presents another promising avenue due to their deregulation in UPS [82]. Pazopanib, a multi-target tyrosine kinase inhibitor, has demonstrated variable efficacy, dependent largely on prior treatments and the specific characteristics of the patient population. Subsequent studies have shown a range of progression-free survival outcomes, underscoring the need for more tailored therapeutic approaches [28]. Anlotinib, another tyrosine kinase inhibitor, has also shown encouraging results, especially in refractory UPS cases, although its broader application calls for more extensive clinical validation [83].

The vascular component of UPS has been targeted through agents like ontuxizumab, which aims at endosialin expressed in the tumor’s neoplastic and stromal cells. This approach reflects a strategic move to disrupt tumor angiogenesis and offers a glimpse into future therapeutic strategies that might leverage vascular targets for more effective sarcoma management [84].

The hypoxic tumor microenvironment, facilitated by factors such as HIF-1α, significantly influences UPS progression. Agents targeting hypoxia-induced pathways, like the PLOD inhibitors, show potential in curtailing the metastatic capacity of tumor cells [85]. Additionally, addressing the heterogeneous immune landscape within UPS tumors, particularly through PD-1/PD-L1 inhibitors like Pembrolizumab, is gaining traction [86]. However, the effectiveness of such therapies can be limited by factors like the IDO1 pathway, which may necessitate combined modality treatments to overcome immune resistance [78].

Research into the cell signaling pathways and cell cycle regulation in UPS has paved the way for the use of phosphoinositide 3-kinase (PI3K) inhibitors, which, when combined with agents like doxorubicin, have significantly delayed tumor growth [87]. The exploration of other targets, such as Neurotensin Receptor 1 (NTSR1) and Fibroblast Growth Factor 23 (FGF23), continues to provide new insights into potential therapeutic strategies that could impede the aggressive behavior and proliferation of UPS cells [29,88].

### 3.17. Prognostic Factors and Survival Outcomes in Undifferentiated Pleomorphic Sarcoma

The prognosis of UPS varies significantly depending on several key factors. The site of the tumor notably influences survival rates, with a 5-year survival rate exceeding 70% for tumors located in the trunk and extremities, compared to less than 50% for those in the head and neck region [89]. This variation may be attributed to the denser blood supply and critical anatomical structures in the head and neck area that complicate surgical intervention and potentially increase the likelihood of residual disease and recurrence.

The American Joint Committee on Cancer (AJCC) staging system is instrumental in assessing the clinical prognosis of UPS, emphasizing the importance of tumor size, depth of invasion, and margin status [90]. Tumors larger than 5 cm, tissue infiltration deeper than 5.5 mm, and positive surgical margins are strongly associated with recurrence, which occurs in more than 30% of patients [91]. Additionally, the presence of a “tail sign” in preoperative imaging is indicative of a high risk for recurrence [49]. Factors influencing the risk of distant metastases include larger tumor size (>2 cm) and invasive characteristics, such as penetration of subcutaneous fat and lymphatic vessels [92].

Socio-demographic characteristics such as age (over 55 years), gender (male), and ethnicity (white), along with clinical factors like immunosuppression and lymphovascular invasion, are linked to an increased risk of all-cause mortality in UPS patients [93]. Furthermore, patients with radiation-associated undifferentiated pleomorphic sarcoma generally exhibit a worse prognosis [94].

A deep tumor location and AJCC stage are among the most critical prognostic factors for UPS, predicting both the potential for disease recurrence and overall survival [95]. A large tumor size, inadequate surgical margins, and the presence of metastatic spread are additional predictors of poor prognosis [52]. While the majority of undifferentiated sarcomas are high-grade lesions with significant local recurrence and metastatic rates, only a minority of patients develop metastases beyond five years, with the lungs being the most common site of distant disease [52].

### 3.18. Importance of Collaborative Research and Data-Sharing

Collaborative research and data-sharing are vital in advancing our understanding of retroperitoneal UPS and developing effective treatments. Given the rarity of this disease, individual institutions often face challenges in accumulating sufficient cases to conduct statistically significant studies. By pooling resources, data, and expertise from multiple institutions and researchers, collaborative efforts enhance the capacity to conduct comprehensive and meaningful research.

Collaborative research initiatives facilitate the identification and validation of biomarkers, enabling better diagnostic and prognostic tools. Moreover, these collaborations support the evaluation of novel therapies and treatment protocols, increasing the likelihood of discovering effective interventions. Data-sharing among researchers and institutions also accelerates progress by reducing duplication of efforts, allowing for more efficient use of resources, and fostering innovation through diverse perspectives.

International consortia and research networks dedicated to sarcoma research exemplify the benefits of such collaborations. These networks can lead to the development of large, multi-institutional databases that provide invaluable insights into the disease’s molecular pathogenesis and treatment responses.

### 3.19. Comprehensive Summary of Retroperitoneal UPS Characteristics

As we delve into the complexities of retroperitoneal undifferentiated pleomorphic sarcoma, the critical aspects of its epidemiology, clinical presentation, diagnostic challenges, and therapeutic strategies have been systematically summarized in Table 1, This table serves as an accessible reference for healthcare professionals and researchers to better understand and manage this rare and aggressive sarcoma, reflecting the comprehensive insights gathered throughout this study.

## 4. Conclusions

In conclusion, this comprehensive study provides a detailed analysis of retroperitoneal undifferentiated pleomorphic sarcoma (UPS), aiming to broaden our understanding of its epidemiological, histopathological, and therapeutic dimensions. Despite its rarity and the diagnostic challenges it poses due to its aggressive and often ambiguous presentation, effective management of UPS requires a multidisciplinary approach that integrates advanced imaging, precise surgical techniques, and individualized treatment plans, including radiation and chemotherapy. Our case study highlights the importance of surgical intervention in achieving disease control and underscores the necessity for ongoing research to refine therapeutic strategies and improve patient outcomes. Moreover, the exploration of new molecular and genetic pathways in UPS opens potential avenues for targeted therapies, promising enhancements in the prognostic evaluation and treatment of this formidable disease.

## Figures and Tables

**Table 1:** Key characteristics of retroperitoneal undifferentiated pleomorphic sarcoma.

Characteristic	Description
Incidence and Prevalence	Rare, accounting for about 1–2% of all solid tumors. Retroperitoneal occurrence is particularly uncommon.
Age of Onset	Predominantly affects middle-aged and elderly males, presenting in the sixth to seventh decades of life.
Symptoms	Indirect and related to the tumor’s impact on surrounding tissues, including nonspecific abdominal pain, weight loss, and anemia.
Diagnostic Challenges	Diagnosing UPS is complex due to its rarity and nonspecific imaging characteristics. Requires exclusionary diagnostic methods often involving advanced imaging like CT and MRI.
Histopathological Features	Displays a complex and highly variable architecture with a predominance of spindle, pleomorphic, and epithelioid cell types. Frequent features include necrosis, hemorrhage, and a high number of mitotic figures.
Molecular and Genetic Profile	Characterized by a variety of genetic and epigenetic alterations. Notable for the presence of TP53 mutations, deletions, and other chromosomal aberrations contributing to tumor behavior.
Treatment Approaches	Primarily surgical resection with an emphasis on achieving negative margins due to the high risk of recurrence and metastasis. Radiation and chemotherapy are used based on tumor location and stage.
Prognosis and Survival Factors	Prognosis varies significantly based on tumor size, location, and surgical outcomes. Deep-seated tumors and inadequate surgical margins are associated with poorer prognosis.

## Data Availability

This article, being a case report and literature review, does not contain original primary data. The information presented is derived from previously published sources and the patient’s medical records.
